# Resilience in Emergency Medicine during COVID-19: Evaluating Staff Expectations and Preparedness

**DOI:** 10.3390/jpm13111545

**Published:** 2023-10-28

**Authors:** Mariusz Goniewicz, Anna Włoszczak-Szubzda, Ahmed M. Al-Wathinani, Krzysztof Goniewicz

**Affiliations:** 1 Department of Emergency Medicine, Medical University of Lublin, 20-081 Lublin, Poland; 2 Faculty of Human Sciences, University of Economics and Innovation, 20-209 Lublin, Poland; anna.wloszczak-szubzda@wsei.lublin.pl; 3 Department of Emergency Medical Services, Prince Sultan Bin Abdulaziz College for Emergency Medical Services, King Saud University, Riyadh 11451, Saudi Arabia; ahmalotaibi@ksu.edu.sa; 4 Department of Security, Polish Air Force University, 08-521 Deblin, Poland; k.goniewicz@law.mil.pl

**Keywords:** COVID-19, healthcare professionals, organizational expectations, personal protective equipment (PPE), post-traumatic stress disorder (PTSD), workplace safety, epidemic preparedness, debriefing

## Abstract

Introduction: The COVID-19 pandemic brought about significant challenges for health systems globally, with medical professionals at the forefront of this crisis. Understanding their organizational expectations and well-being implications is crucial for crafting responsive healthcare environments. Methods: Between 2021 and 2022, an online survey was conducted among 852 medical professionals across four provinces in Poland: Mazovia, Łódź, Świętokrzyskie, and Lublin. The survey tool, based on a comprehensive literature review, comprised dichotomous questions and specific queries to gather explicit insights. A 5-point Likert scale was implemented to capture nuanced perceptions. Additionally, the Post-Traumatic Stress Disorder Checklist-Civilian (PCL-C) was utilized to ascertain the correlation between workplace organization and post-traumatic stress symptoms. Results: A noteworthy 84.6% of participants believed their employers could enhance safety measures, highlighting a discrepancy between healthcare workers’ expectations and organizational implementations. Major concerns encompassed the demand for improved personal protective equipment (44.6%), structured debriefing sessions (40%), distinct building entrances and exits (38.8%), and psychological support (38.3%). Statistical analyses showcased significant variations in ‘Avoidance’ and ‘Overall PTSD Score’ between individuals who had undergone epidemic safety procedure training and those who had not. Conclusions: The results illuminate the imperative for healthcare organizations to remain agile, attentive, and deeply compassionate, especially during worldwide health emergencies. Despite showcasing remarkable resilience during the pandemic, medical professionals ardently seek an environment that underscores their safety and mental well-being. These findings reinforce the call for healthcare institutions and policymakers to champion a forward-thinking, employee-focused approach. Additionally, the data suggest a potential avenue for future research focusing on specific demographic groups, further enriching our understanding and ensuring a more comprehensive readiness for impending health crises.

## 1. Introduction

The COVID-19 pandemic, which began in late 2019, swiftly transformed into a global health crisis, significantly impacting the dynamics of healthcare systems worldwide [1]. As emergency departments bore the brunt of rising case numbers, the on-ground staff—comprising nurses, medical rescuers, doctors, and healthcare caregivers—faced an unprecedented surge in patient volume and acuity [2]. In these challenging circumstances, the expectations and safety concerns of these medical professionals regarding organizational support emerged as pivotal in ensuring the efficient management of the crisis.

Central to this narrative was the relationship between healthcare professionals and their employers. While literature [3,4,5,6] emphasizes the importance of protective equipment, adequate training, and psychological support for healthcare workers during pandemics, how effectively were these requirements met during the COVID-19 crisis? Moreover, what were the tangible and immediate expectations of these professionals, and how did they align with organizational provisions? A failure to address these questions and concerns in real time may lead to increased vulnerabilities, not only for healthcare workers but also for the patients relying on them.

Adding a layer of immediacy to this discourse is the realization that the challenges thrown up by the pandemic are not merely localized. Instead, the implications of these challenges resonate on a global scale [7]. From a hospital in New York to a clinic in New Delhi, the narrative of medical professionals battling the pandemic, armed with their skills, resilience, and expectations from their employers, remains a shared story.

The universal impact of the COVID-19 pandemic was felt in every corner of the world. However, the organizational responses to these challenges displayed significant regional variations, influenced by a myriad of factors ranging from governmental policies to available resources and the socio-economic landscape.

For instance, countries like South Korea and New Zealand quickly became models of effective employer support [8,9]. In South Korea, medical professionals were promptly provided with an abundance of personal protective equipment (PPE), and a robust testing system was put in place [10]. This not only protected the healthcare workers but also ensured that patients received timely and safe treatment. Meanwhile, in New Zealand, clear communication between healthcare employers and their staff, coupled with the government’s early action, significantly alleviated the pressures faced by frontline workers [11].

Conversely, in other regions, medical professionals battled not only the virus but also systemic issues, ranging from a lack of adequate PPE to insufficient training on the ever-evolving treatment protocols. For many, their organizational expectations were rooted more in hope than in the assurance of support [12,13,14].

These regional disparities underscore the importance of understanding the diverse organizational expectations of medical professionals across the globe. By highlighting best practices and learning from the challenges faced in various regions, the global healthcare community can foster a more collaborative and effective response to future health crises.

Given the intricate web of global challenges and the varied responses of healthcare systems worldwide, there is an undeniable need to scrutinize the specific expectations medical professionals held during the peak of the pandemic. This examination goes beyond simply documenting reactions; it seeks to inform and better prepare healthcare systems for potential future crises.

This research aims to elucidate the organizational expectations of medical professionals during the COVID-19 pandemic, with a focus on its implications for emergency medicine. By analyzing variations among different healthcare settings and evaluating if these expectations were met, we hope to foster a global discourse on strengthening healthcare systems for future emergencies.

## 2. Materials and Methods

### 2.1. Location of the Study

The research was conducted from 2021 to 2022. Owing to the constraints of the pandemic, the study was executed online, with the survey link disseminated to medical facilities in four provinces of Poland: Mazovia, Łódź, Świętokrzyskie, and Lublin. The choice of these specific provinces was multi-faceted. They were strategically chosen to provide a balanced geographical representation, capturing key regions of the country. Additionally, historical epidemiological trends and healthcare engagement levels in these provinces demonstrated a level of consistency that dovetailed neatly with our research objectives. The logistical feasibility of focusing on these areas, especially during the challenges posed by the pandemic, ensured streamlined communication with local medical facilities and professionals. Furthermore, pre-existing collaborations and contacts within these provinces’ medical communities facilitated the efficient distribution and management of the survey, ensuring that we acquired a comprehensive yet representative sample of healthcare professionals.

### 2.2. Study Population

The study encompassed 852 medical professionals from diverse healthcare settings. While the participants represented a variety of roles, including paramedics, doctors, and medical caregivers, a significant majority (82.6%) identified as nurses. The gender distribution was notably skewed, with females constituting 88.1% of the cohort. The diverse array of healthcare entities from which these professionals hailed includes Primary Healthcare, Specialist Ambulatory Care, Emergency Departments (ED), Care and Treatment Institutions, Social Welfare Homes, and various hospital departments. These departments differentiated themselves based on the nature of the patients they catered to—either primarily those diagnosed with conditions other than COVID-19 or those suspected/confirmed as having COVID-19. Additionally, the study included Ambulance teams, which were categorized based on their dispatch specifics—catering either to non-suspected or suspected/confirmed COVID-19 patients.

### 2.3. Questionnaire

The survey was meticulously designed to capture a holistic understanding of the healthcare professionals’ experiences during the pandemic. It commenced with collecting demographic data, which was pivotal in understanding the diverse backgrounds and expertise of the respondents. This section included items such as age, gender, years of experience, and specific roles within the healthcare sector.

Subsequent sections dived deeper into the main crux of the research. These segments aimed to excavate the expectations and perceptions healthcare workers harbored regarding their employers’ role in organizing work amidst the pandemic. To ensure comprehensive coverage of opinions and experiences, a mix of question types was used. Dichotomous questions provided clear binary perspectives, while open-ended queries allowed respondents to share detailed insights. Moreover, to capture the nuanced gradations of opinions, a 5-point Likert scale was incorporated, ranging from ‘Strongly Disagree’ to ‘Strongly Agree’, providing a spectrum of agreement for various statements.

A robust foundation for the survey was laid by a literature review. This entailed a systematic investigation of published works that touched upon work organization during the pandemic and medical personnel’s associated expectations. It ensured that our tool was grounded in existing research while addressing gaps and evolving challenges.

However, a survey is not just about questions; its efficacy lies in its clarity and relevance. To this end, we employed a qualitative method to verify the tool’s effectiveness. A pilot test was conducted on a sample of 10 individuals from diverse medical backgrounds. Their primary role was to evaluate the clarity, relevance, and comprehensiveness of our questions. Feedback from this initial group was instrumental, highlighting areas for refinement. It is worth noting that to maintain data purity, the results from these dual-role participants were deliberately kept separate, ensuring that they did not influence the main study’s findings.

Furthermore, in our bid to cement the tool’s credibility, we subjected the survey to the scrutiny of expert judges. These individuals, with their vast academic and practical experience, evaluated our questionnaire for clarity, comprehensiveness, and relevancy to the current pandemic backdrop.

Additionally, understanding the potential psychological impact of the pandemic on healthcare workers was vital. Thus, we incorporated the PCL-C into our research. This standardized tool is recognized for its accuracy in gauging post-traumatic stress disorder (PTSD) symptoms. With its inclusion, we aimed to establish any correlations between the organization and expectations in medical units with the manifestation of PTSD symptoms among healthcare professionals.

### 2.4. Statistical Analysis

To answer the research questions and test the hypotheses, statistical analyses were conducted using the IBM SPSS Statistics software, version 26. Basic descriptive statistics, Pearson’s r correlation analyses, independent *t*-tests, and Mann–Whitney U tests were performed. A classic threshold of α = 0.05 was adopted for statistical significance.

### 2.5. Ethical Considerations

While the study does not qualify as a medical experiment under Polish law and thus did not necessitate a Bioethics Committee’s oversight, it still followed rigorous ethical standards. Participants were briefed about the research objectives, assured of confidentiality, and reminded of their participation’s voluntary nature. Data storage protocols adhered to strict privacy guidelines, and informed consent was acquired from all participants.

## 3. Results

Our study had strong representation from females, who made up 88.1% of the participants, while males accounted for only 11.9%. This gender and professional role skewness, particularly the overrepresentation of nurses (82.6%), underscores a specific demographic dynamic in our data collection, which we have further elucidated in the limitations section. The age range of the participants spanned from 20 to 59 years, averaging at 39 years. In terms of professional experience, participants reported an average of 12 years, with durations as brief as a month and as lengthy as 41 years.

Diving deeper into their job roles, after the prominent presence of nurses (82.6%), paramedics followed at 8.9%. The dataset recorded fewer representations from professions like medical caregivers and physicians, with other job roles constituting the remaining 6% of responses.

When exploring their professional involvement during the SARS-CoV-2 pandemic, a significant 91.8% affirmed their active participation in healthcare duties. The primary work setting for 40.3% was non-COVID-19 hospital wards. In contrast, 21.9% were deployed in COVID-designated wards. Other crucial segments included 14% stationed in primary healthcare and 14.4% working from unspecified locations. Interestingly, nearly one in five participants disclosed affiliations with more than one workplace, as detailed in Table 1.

### 3.1. Training and Pandemic Preparedness

Upon exploring their exposure to training, we discerned that a considerable majority (73.2%) had received training at their respective workplaces, specifically targeting adherence to safety measures during epidemics/pandemics. Contrarily, 26.8% lacked such training. Further insight into the facilities’ pandemic preparedness revealed that participants, on average, rated their workplace’s readiness level at 3.19 (with a standard deviation of 0.97). A moderate level of preparedness was the consensus for 43.8% of the respondents. These findings are detailed in Table 2.

### 3.2. Evaluation of Employers’ Safety Measures

Participants evaluated the safety measures implemented by their employers during the pandemic. A striking 84.6% believed that their employers had potential areas of improvement regarding the implementation of safety measures. In contrast, 15.4% felt that their employers had already maximized safety precautions. When discussing improvements, a significant 44.6% emphasized the need for an enhancement in both the quality and quantity of personal protective equipment provided. Close behind, about 40% highlighted the importance of organized debriefings. Such sessions would allow staff to collaboratively discuss challenging situations, identify areas of concern, and refine their approach. In a similar vein, 38.8% believed that establishing distinct building entry and exit points for personnel directly attending to COVID-19 patients would bolster safety by segregating them from the rest of the staff. Additionally, 38.3% underscored the critical need for psychological support, suggesting that access to counseling and related services was indispensable during these challenging times (Table 3).

### 3.3. PTSD Symptoms and Their Association with Training and Preparedness

Our investigation illuminated a dichotomy in PTSD symptomatology predicated on participants’ training pedigree. Specifically, individuals devoid of prior training on epidemic safety protocols exhibited heightened symptoms in the ‘Avoidance’ and ‘Overall PTSD Score’ metrics. It is intriguing to note that parametric tests affirmed these differences as statistically significant. However, when these findings were juxtaposed against the results from the non-parametric Mann–Whitney U test, the differences in the aforementioned indices were not mirrored. Notably, for other scales, such as ‘Intrusion’ and ‘Hyperarousal’, the dichotomy remained elusive, with no discernible differences arising from either testing methodology. Further, a correlation analysis revealed a negative, albeit weak, relationship between PTSD symptoms and the assessment of an organization’s pandemic preparedness. This indicates that as the perceived preparedness of the workplace increased, the intensity of PTSD symptoms reported by participants decreased (Table 4).

## 4. Discussion

The findings of our study shed critical light on the organizational expectations of medical professionals during the height of the COVID-19 pandemic, especially in the context of emergency medicine. A majority (84.6%) believed that their employer could further enhance safety measures, pointing towards a perceptual gap between healthcare workers’ expectations and organizational practices. Such a significant percentage underscores the necessity for institutions to continually evaluate and adapt their safety measures in line with the evolving needs of their staff.

While our study had a dominant representation of female participants, it is essential to understand the unique challenges and perspectives brought by gender dynamics in healthcare during the pandemic. It is also worth noting that perceptions related to healthcare delivery, especially in emergency settings, may vary significantly based on factors such as gender and education. For instance, studies like the one by Tiziana Ciarambino et al. have highlighted that older and less-educated females can have distinct experiences and evaluations of care during the pandemic [15]. Given our study’s significant representation of female professionals, predominantly nurses, further studies should consider these intersections of age, gender, and education to provide a more nuanced understanding of expectations and perceptions in healthcare settings during emergencies.

Previous studies have shown gender-based disparities in stress perception, workload, and even access to protective measures in healthcare settings [16,17]. Additionally, with a varied age range, understanding how different age groups perceived organizational support, especially considering factors like risk perception and familial responsibilities, would offer a richer context.

While the primary focus of our study was on the direct experiences and perceptions of medical professionals, it is crucial to recognize the role of socio-economic disparities.

Beyond socio-economic factors, demographic characteristics, particularly age and education, have been shown to play an integral role in shaping the experiences of medical professionals. It is essential to understand how these dynamics influence perceptions of preparedness, especially when considering the diverse workforce engaged during the pandemic. Such an understanding could provide valuable insights for healthcare institutions and policymakers to tailor strategies that cater to the varied needs and perspectives of their staff.

Medical professionals working in underfunded or resource-scarce institutions may have different expectations and challenges compared to those in better-funded environments. Disparities in funding, infrastructure, and access to resources can lead to varied levels of satisfaction, trust, and even perceptions of safety [18]. Recognizing these socio-economic divides is essential for a holistic understanding of the healthcare landscape during the pandemic.

One of the salient expectations voiced by participants was the desire for improved quality and quantity of personal protective equipment. With 44.6% of respondents emphasizing this, it reaffirms the global narrative surrounding PPE shortages and the resultant vulnerabilities faced by healthcare professionals [19,20]. The shortage of PPE not only jeopardizes the safety of medical staff but can also impact the quality of care provided, potentially exacerbating the health crisis.

Furthermore, the emphasis placed by 40% of the participants on organizing debriefings signals an inherent need for communicative feedback loops within medical institutions. Debriefings provide a platform for collective reflection, error identification, and procedural refinement. Such practices are integral, especially during health emergencies, ensuring the adaptability and resilience of healthcare systems [21,22].

Drawing parallels with previous health crises, such as the SARS or MERS outbreaks, reveals recurring themes in healthcare workers’ expectations. The demand for adequate protective measures, clear communication, and psychological support has been consistent [23,24]. However, the unprecedented scale of the COVID-19 pandemic might have intensified these needs, making it imperative for organizations to learn from the past while innovatively addressing the present challenges.

As the world grappled with the pandemic, technology and digital health emerged as crucial allies. While our study touched upon the tangible needs of healthcare professionals, it might be worth exploring if there was an underlying expectation or desire for better technological support. Enhanced telehealth platforms, digital tracking of resources, or even AI-driven patient management tools could offer additional layers of support and efficiency, reducing the strain on our frontline heroes [25,26].

The pivotal role of leadership during such crises cannot be understated. Strong leadership and transparent communication influence not only the operations but also the morale and confidence of medical professionals [27]. When leadership is decisive, communicative, and supportive, it creates an environment where professionals feel valued, heard, and reassured [28]. The impact of leadership styles and strategies during the COVID-19 pandemic might be an invaluable area for further investigation, especially in understanding its influence on healthcare workers’ perceptions.

The call by 38.8% of participants for separate building entrances and exits also brings forth an essential aspect of infection control, which often goes beyond the immediate scope of personal protection. Such measures, while logistically challenging, can significantly reduce cross-contamination risks, protecting both healthcare workers and patients.

The implications of meeting or failing to meet the expectations of medical professionals stretch beyond their personal well-being. Patient care, the crux of the medical profession, is intrinsically tied to the conditions under which professionals operate [28]. When healthcare workers’ needs are not addressed, it could lead to reduced efficiency, heightened stress, and potential lapses in care quality [29,30]. Addressing the expectations of healthcare professionals is, by extension, a commitment to ensuring optimal patient care and outcomes.

A concerning aspect of our findings is the apparent psychological toll the pandemic has exerted on medical professionals. With 38.3% underscoring the need for psychological support and counseling access, it is evident that the crisis has had profound mental health implications. Previous studies during past health crises have similarly emphasized the psychological vulnerabilities of frontline healthcare workers, necessitating robust mental health support structures [31,32,33,34].

Our study underscores the importance of regular feedback mechanisms. Institutions should consider periodic surveys, town hall meetings, or even anonymous feedback platforms. These mechanisms will ensure that the evolving needs of healthcare workers are promptly addressed, fostering an environment of trust and proactive adaptability.

Our statistical analyses also provide a nuanced perspective on the significance of training. While parametric tests revealed stark differences in ‘Avoidance’ and ‘Overall PTSD Score’ between those trained in safety procedures during epidemics and those not, non-parametric tests did not corroborate this entirely. This dichotomy indicates the multifaceted nature of psychological responses to crises and underscores the need for more comprehensive research in this area.

Our findings are not just significant for medical institutions but hold substantial policy implications. Decision-makers at governmental or institutional levels could benefit from integrating these insights into their policy formulations. Addressing the gaps between healthcare workers’ expectations and the realities on the ground could lead to policies that prioritize not only immediate safety and resources but also long-term well-being and resilience. Ensuring that the voice of the frontline resonates in policy can streamline the response to future health crises and strengthen the healthcare sector at large [35,36].

While our study highlighted the significance of training, it is imperative to delve deeper into the content and quality of these programs. Are our current training modules adequately equipped to handle the unique challenges posed by pandemics like COVID-19? Given the varied psychological responses noted, there might be a need to incorporate a blend of technical, psychological, and crisis-management skills in training regimens. Such a holistic approach could ensure that medical professionals are not just technically adept but also mentally fortified for unprecedented challenges.

In light of the expectations voiced by medical professionals, there are broader implications for healthcare infrastructure. An adaptive response to immediate challenges, such as those posed by the COVID-19 pandemic, offers a blueprint for handling future health crises. By integrating robust feedback mechanisms, addressing the holistic well-being of healthcare workers, and ensuring adequate resources, medical institutions can be better equipped not just to manage crises but to foster a culture of continual improvement and resilience [37]. This proactive approach could also be pivotal in attracting and retaining talent in the healthcare sector, ensuring that our medical institutions remain robust and reliable in the face of any future challenges.

Building upon our findings, healthcare institutions can take proactive measures to bridge the perceptual gap between organizational practices and workers’ expectations.

Firstly, periodic training sessions could be organized that are tailored to the unique challenges of pandemics like COVID-19. These should not only focus on technical skills but also emphasize psychological readiness and coping strategies. Secondly, the establishment of a centralized digital platform can streamline communications, resource tracking, and feedback collection, ensuring that immediate needs and concerns are promptly addressed. Recognizing socio-economic disparities, resource allocation should be prioritized for underfunded institutions to ensure equity in safety measures and care quality. Lastly, fostering transparent communication from leadership can be achieved through regular updates, town hall meetings, and open forums, ensuring that all staff members feel valued, informed, and heard. These suggestions, when implemented, could lead to an environment that is not only safer but also more supportive for healthcare workers during global health emergencies.

Moreover, understanding the intricate role of demographics, particularly age, gender, and education, can help institutions develop tailored programs and interventions. For example, specific training modules for older professionals or those with varied educational backgrounds could be beneficial. Recognizing and addressing these nuances ensure that all medical professionals, irrespective of their demographic backgrounds, feel adequately supported and prepared.

Based on the insights from this study, there is a compelling case for further research that narrows its focus to specific demographic groups. Investigating the perceptions of older and less-educated females concerning healthcare delivery and PTSD symptoms during the COVID-19 pandemic could offer valuable contributions to the field.

In sum, our findings provide a holistic insight into the organizational expectations of medical professionals during the COVID-19 pandemic. The study reiterates the importance of adaptive, responsive, and supportive organizational structures, especially during global health emergencies. Ensuring that these expectations are met is not just about securing the present but also about fortifying the future, preparing healthcare systems for similar challenges that lie ahead.

## 5. Limitations

Our study, while comprehensive in its approach, bears certain limitations that are crucial to acknowledge. First and foremost, the geographical focus on four specific provinces in Poland might not encapsulate the broader experiences and expectations of medical professionals throughout the country or in varied global settings. The unique characteristics of these provinces could introduce biases not present in other areas, and thus, extrapolation to a larger context requires caution. The demographic makeup of our participants is heavily skewed towards female representation, with a dominant 88.1% being women. Moreover, a significant 86.6% of our respondents identified as nurses. This raises concerns about the generalizability of our findings, as the experiences and expectations of male healthcare professionals and other medical roles might be underrepresented or overlooked entirely in our dataset. This overrepresentation could inadvertently lead to a bias in our findings that primarily echo the experiences and concerns of nurses, particularly female nurses.

An important limitation to note is the noticeable underrepresentation of physicians in our study. While nurses formed a significant majority of respondents, physicians—key medical decision-makers during the pandemic—were limited in number. Several factors could explain this discrepancy: physicians’ intense workloads during the pandemic might have hindered participation, the channels used for survey dissemination might not have reached physicians as effectively, or there might be a difference in survey participation inclination between professions. This lack of physician perspectives could affect the depth of insights drawn, especially relating to decision-making and organizational expectations during the crisis.

Moreover, we did not stratify our findings based on education levels or specific age brackets, particularly among older participants. Existing literature, including the study by Tiziana Ciarambino et al., indicates that perceptions related to healthcare delivery, particularly in emergency settings during the COVID-19 pandemic, can vary significantly based on demographic factors such as age and education. Specifically, older and less-educated females might have distinct perceptions that could deviate from the general trend identified in our research. By not considering these stratifications, we might be overlooking critical nuances in the experiences and perceptions of certain demographic subgroups.

The decision to conduct the research exclusively online—a necessity given the pandemic restrictions—might have introduced a selection bias. This mode could exclude potential respondents who might have been more accessible or responsive to traditional survey methodologies. Furthermore, given the emotionally charged nature of the subject, there was potential for response bias. Participants with particularly strong opinions or feelings, whether positive or negative, might have been more inclined to respond, possibly sidelining more neutral or indifferent perspectives.

Another aspect worth noting is the scope of our inquiry. While our questionnaire was thorough and rooted in existing literature, it is conceivable that certain nuanced or intangible facets of participants’ experiences were not fully explored. This can always be a challenge in quantitative research, where open-ended, qualitative insights are limited. Lastly, the timeline of the pandemic introduced the possibility of recall bias. As participants reflected on experiences spanning a significant period, their memories of events or feelings from earlier stages might not have been entirely precise.

## 6. Conclusions

Surveying 852 medical professionals across four provinces in Poland, our study offers compelling insights into their organizational expectations during the pandemic. A clear majority voiced concerns about workplace safety, emphasizing the necessity for enhanced protective measures, distinct facility access routes, structured debriefings, and bolstered psychological support. The notable correlation between adequate safety training and decreased post-traumatic symptoms further underscores the importance of professional training during such crises.

These findings resonate beyond Poland’s borders, encapsulating a global plea for healthcare establishments to be adaptable, attentive, and deeply empathetic. Medical professionals have showcased tremendous dedication and adaptability, and in turn, they seek an environment that prioritizes their safety, acknowledges their contributions, and upholds their mental well-being.

One area for further exploration, as suggested by the data, is to delve deeper into the experiences of specific demographic groups, particularly older and less-educated females. Their perceptions concerning healthcare delivery and PTSD symptoms during pandemics like COVID-19 could offer additional layers of understanding and contribute significantly to the broader knowledge base.

In anticipating future health challenges, it is crucial for global healthcare entities and leaders to internalize and act on these insights. An anticipatory, worker-centric approach, coupled with targeted research initiatives, can invigorate the medical community and fortify our health infrastructures against subsequent challenges.

In essence, our study underscores the profound significance of active listening, timely adaptation, and unwavering support. As the world navigates towards recuperation, embracing these tenets will be central to forging a more inclusive, resilient, and compassionate tomorrow.

## Figures and Tables

**Table 1:** Demographic data.

Gender	N	%
Women	751	88.1
Men	101	11.9
Age (average ± SD)		
39.02 ± 10.02 years (20–59 years)		
Work experience (average ± SD):		
12.90 ± 11.44 years (0.08–41 years)		
Profession:	N	%
Nurse	704	86.6
Paramedic	75	8.9
Physician	8	0.9
Medical caregiver	14	1.6
Other	51	6.0
Workplace:	N	%
Primary Healthcare	119	14
Specialist Ambulatory Care	34	4.0
Emergency Department	70	8.2
Admissions Room	47	5.5
Care and Treatment Institution	39	4.6
Social Welfare Home	36	4.2
Hospital, non-COVID-19 ward	343	40.3
Hospital, COVID-19 ward	187	21.9
Ambulance, non-COVID cases	36	4.2
Ambulance, COVID cases	48	5.6
Other	123	14.4
Worked in healthcare during the pandemic?	N	%
Yes	782	91.8
No	70	8.2
Total	852	100

SD means standard deviation; N means the total number of individuals or observations in the sample.

**Table 2:** Assessment of Pandemic Preparedness, Training, and Employee Safety Assurance.

Question/Category	N	%
Underwent safety training during pandemic		
Yes	624	73.2%
No	228	26.8%
Organization’s pandemic preparedness level		
1—Unprepared	38	4.5%
2	141	16.5%
3—Neutral	373	43.8%
4	220	25.8%
5—Fully Prepared	80	9.4%
Employer could improve in ensuring employee safety		
Yes	721	84.6%
No	131	15.4%
Total respondents	852	100%

**Table 3:** Assessment of Employer Effectiveness in Ensuring Employee Safety and Suggested Improvements.

Questions/Measures	N	%
Employer’s Effectiveness in Ensuring Employee Safety		
Could your employer improve in ensuring employee safety?		
Yes	721	84.6%
No	131	15.4%
Suggested Measures for Improving Employee Safety *		
Reorganize work structure	42	4.9%
Provide separate entrances and exits for staff directly working with COVID-19 patients and other personnel	331	38.8%
Ensure a higher quantity and quality of personal protective equipment	380	44.6%
Provide employees with psychological support, contact with a psychologist	326	38.3%
Organize debriefings to discuss challenging situations, assess mistakes, and refine procedures	341	40.0%
Other measures not mentioned in the survey	34	4.0%
Total respondents	852	100%

* This mean multiple answers were allowed.

**Table 4:** PTSD Symptoms in Relation to Training Participation and Organizational Preparedness for Pandemics.

Measures/Criteria	Trained (n = 624)	Not Trained (n = 227)	r Pearson [Org. Preparedness Rating]	Significance
Intrusion	M: 9.94, SD: 3.73	M: 10.57, SD: 4.35	−0.16	<0.001
Avoidance	M: 14.79, SD: 5.52	M: 15.73, SD: 6.02	−0.17	<0.001
Hyperarousal (Increased Arousal)	M: 12.58, SD: 4.57	M: 13.15, SD: 4.73	−0.18	<0.001
PTSD—Overall Score	M: 37.30, SD: 12.44	M: 39.44, SD: 13.92	−0.19	<0.001

Note: The *t*-test results for ‘Avoidance’ and ‘Overall Score’ were statistically significant with weak effects. This highlights higher PTSD symptom intensity among those who did not receive training compared to those who did. However, the Mann–Whitney U test did not show significant differences for these indices. Importantly, both tests found no differences for the ‘Intrusion’ and ‘Hyperarousal’ measures. The correlation analysis indicated that as the preparedness rating of the workplace for a pandemic increased, the PTSD symptoms’ intensity experienced by participants decreased. This suggests that training and preparedness can influence PTSD symptoms among professionals.

## Data Availability

The datasets used and/or analyzed during the current study are available from the corresponding author on reasonable request.
